# Melatonin and/or rowatinex attenuate streptozotocin-induced diabetic renal injury in rats

**DOI:** 10.7555/JBR.31.20160028

**Published:** 2019

**Authors:** Tarek K Motawi, Samia A Ahmed, Manal A Hamed, Shohda A El-Maraghy, Wessam M Aziz

**Affiliations:** 1. Biochemistry Department, Faculty of Pharmacy, Cairo University, Cairo, Egypt; 2. Therapeutic Chemistry Department, National Research Centre, Dokki, Cairo, Egypt.

**Keywords:** diabetic nephropathy, melatonin, rowatinex, histopathology

## Abstract

The study aimed to explore the prophylactic effect of melatonin, rowatinex; a naturally occurring renal drug, and its combination on diabetic nephropathy in type 2 diabetic rats. Diabetes was induced by intraperitoneal injection of a single dose of streptozotocin (50 mg/g body weight). Three days before diabetes induction, rats were daily treated with melatonin, rowatinex and their combination continuously for 8 weeks. Evaluation was done through measuring blood urea nitrogen (BUN), serum uric acid, serum creatinine, urine creatinine, creatinine clearance, nitric oxide (NO), malondialdehyde (MDA), superoxide dismutase (SOD), glutathione (GSH), total antioxidant capacity (TAC), kidney injury molecule-1 (KIM-1), heat shock protein-70 (HSP-70), caspase-3, transforming growth factor β1 (TGFβ1), DNA degradation by the comet assay and total protein contents. Histopathologic study was also done for the kidney and the pancreas. Drastic changes in all measured parameters of the diabetic rats were observed. Treatment with melatonin and rowatinex showed amelioration to variable degrees. In conclusion, melatonin showed the most potent effect on protecting rats from deleterious action of diabetic nephropathy followed by its combination with rowatinex.

## Introduction

Diabetes is a disease with many complications and characterized by chronic hyperglycemia that may lead to microvascular complications, including nephropathy and macrovascular complications if left untreated [^1^]. The kidney is a vital organ that removes waste products of tissue metabolism through the plasma into the urine and maintains homeostasis of essential cellular biomolecules [^2^]. During diabetic nephropathy, increase in type IV collagen and decrease in heparan sulfate (HS) are known to take place [^3^]. Type IV collagen is one of the constituents of the extracellular matrix (ECM) in the glomerular basement membrane (GBM) that provides a scaffold for other ECM components by its network-like structure [^3^]. The thickening of GBM and the alteration of its composition during diabetic nephropathy lead to abnormal filtration [^4^].

Melatonin (*N*-acetyl-5-methoxytryptamine) is a pineal gland hormone. It has a variety of physiologic, immunological and biochemical functions. It is an endogenous free-radical scavenger and exerts chemoprotective, immunostimluatory and myelostimulatory effects [^8^], which are important in the treatment of diabetic complications [^9^]. Moreover, melatonin interacts with insulin [^10^], and consequently alleviates the adverse effects of diabetes by decreasing blood glucose levels and enhancing antioxidant defense capacities [^11^] to detoxify harmful reactive oxygen species [^10^]. Melatonin has been shown to decrease the amount of superoxide radicals in rat plasma, increase total antioxidative capacity and antioxidative enzymes including superoxide dismutase and glutathione peroxidase [^10^].

Rowatinex is a pharmaceutical product containing naturally occurring terpenes (31% pinene, 15% camphene, 10% borneol, 4% anethole, 4% fenchone, and 3% cineole). After oral intake, rowatinex is rapidly absorbed due to the presence of terpenes which are lipid-soluble substances. The hydrocarbons in pinene participate in similar pathways of absorption, metabolism and excretion of polar oxygenated metabolites [^12^].

The aim of the present study was to explore the effects of melatonin, rowatinex and their combination on streptozotocin-induced diabetic nephropathy in rats.

## Materials and methods

### Chemicals

All chemicals used were of high analytical grade and purchased from Merck, Germany and Sigma, USA.

### Animals and ethics

Eighty male Wistar albino rats (150-200 g) were obtained from the Animal House of the National Research Centre, Dokki, Giza, Egypt. Rats were fed on a standard diet with free access to tap water. They were kept for two weeks to be acclimatized to the environmental conditions. The study protocol was approved by the local institutional review board at the authors’ affiliated institutions and animal studies were carried out in accordance with the established institutional guidelines regarding animal care and use and strictly in accordance with the Medical Ethical Committee of the National Research Centre in Egypt (Approval No.: 11063, 4-6-2014).

### Animal studies

The rats were divided into 8 groups (10 rats each). Group 1 rats were orally given normal saline, and group 2, 3 and 4 rats received rowatinex, melatonin and their mixture, respectively. Group 5, 6 and 7 rats were prophylactically treated with rowatinex, melatonin or their combination, respectively, for three days before streptozotocin induction and continuously administered for two months. Normal rats were intraperitoneally administered with melatonin at a dose of 200 µg/ kg body weight [^15^] and rowatinex (Amoun Co., Egypt) was administered by oral gavage at a dose of 300 mg/kg body weight [^16^] for 3 days before diabetes induction. For establishment of the rat diabetes model, rats were injected intraperitoneally with a single dose of streptozotocin (50 mg/kg body weight) dissolved in 0.01 mol/L citrate buffer immediately before use ^[13]^. After injection, the rats had free access to food and water and were given 5% glucose solution to drink overnight to counter hypoglycemic shock [^14^]. After three days of streptozotocin induction, hyperglycemic rats (460–500 mg/dL) were used for the experiment. After 3 days of diabetes induction, the diabetic rats (460-500 mg/dL) were continuously administered with the selected dugs for 8 weeks.

### Sample preparations

At the end of the experiment, the rats were fasted over night and kept in the metabolic cages for urine collection. The urine samples were centrifuged at 3,000 rpm for 10 minutes and the supernatants were stored at -80°C for further determinations.

Blood samples were taken from each rat via the sublingual vein into sterilized tubes and let stand for 10 minutes to clot. The serum was separated by centrifugation at 3,000 rpm for 10 minutes and used for biochemical analysis.

After blood collection, all rats of each group were sacrificed under ether anesthesia. The kidneys from different experimental normal and diabetic groups were removed immediately, weighed and homogenized in 0.9N sodium chloride (1:10 w/v), and centrifuged at 4,000 rpm for 15 minutes. The supernatant was collected and liquated in Epindorff tubes and stored at -20°C for different biochemical determinations.

### Biochemical analysis

The kidney function indices, including blood urea nitrogen (BUN), uric acid, creatinine and creatinine clearance, were estimated using biodiagnostic kits (Biogamma, Stanbio, Germany). The nephropathy indices, including kidney injury molecule (KIM-1), heat shock protein 70 (HSP 70), caspase-3 and transforming growth factor-β1 (TGFβ1), were determined by using ELISA kit (R&D, Minneapolis, MN, USA). The oxidative stress markers which included malondialdehyde (MDA), glutathione (GSH), superoxide dismutase (SOD) and nitric oxide (NO) were detected by the methods of Buege and Aust [^17^], Moron *et al.* [^18^], Nishikimi *et al.* [^19^], and Moshage *et al. *[^20^], respectively. The total antioxidant capacity (TAC) was estimated by using biodiagnostic kits (Stanbio). Serum and kidney total protein contents were estimated by the method of Bradford [^21^]. DNA fragmentation indices were done using comet assay technique.

### Histopathologic analysis

Kidney and pancreas sections of all groups were stained with hematoxylin and eosin (H & E) to detect changes in cell architecture and degree of fibrosis [^22^].

### Statistical analysis

All data were expressed as mean±SD of 10 rats in each group. Statistical analysis was carried out by one-way analysis of variance (ANOVA) accompanied by Costat Software Computer Program using least significant difference (LSD) between groups at *P*<0.05.

## Results

### Biochemical determinations

Concerning body weight, diabetic rats showed a significant decrease by 17.40% as compared with the normal control rats. Rats prophylactically treated with rowatinex, melatonin, or their combination showed significant increase in body weight by 10.50%, 14.11% and 16.47%, respectively, as compared with the diabetic group. Therefore, body weight was ameliorated by 8.73%, 11.65% and 13.59%, respectively available online).

After streptozotocin injection, the glucose levels increased by 543.00% in the diabetic rats vs. the control group and by 528.00% att the end of the experiment. Prophylactic rowatinex, melatonin or their combination reduced the glucose levels by 54.50, 68.60 and 67.20%, respectively, vs. the diabetic rats. Therefore, the glucose level recorded improving levels reached to 341.57%, 431.46% and 421.34%, respectively available online).

BUN significantly increased by 133.00% in the diabetic rats vs. the control group (***Table 1***). Prophylactic rowatinex, melatonin or their combination significantly decreased BUN by 49.70%, 56.80% and 40.70%, respectively, vs. the diabetic group. Therefore, BUN level was ameliorated by 116.00%, 132.00% and 95.00%, respectively. Serum uric acid level in diabetic rats was significantly increased by 196.00%. In diabetic rats treated with rowatinex, melatonin and their combination, the uric acid levels were significantly decreased by 47.50%, 40.30% and 40.40%, respectively, as compared with the diabetic group. Hence, we noticed the improvement by 141.00%, 128.00% and 119.80%, respectively. In case of serum creatinine level, the diabetic rats showed a significant increase by 535.00% vs. the normal control group. Prophylactic rowatinex, melatonin or their combination significantly decreased serum creatinine by 77.30%, 81.90% and 80.00% vs. the diabetic group, respectively. The prophylactic action with these drugs improved the level of serum creatinine by 492.85%, 521.42% and 500.52%, respectively. Regarding to creatinine level in urine, diabetic rats showed a significant decrease by 67.40%. In diabetic rats treated with rowatinex, melatonin and their combination, the serum uric acid levels were significantly increased by 146.00%, 234.60% and 200.00%, respectively, as compared with the diabetic group. Therefore, protection of diabetic rats with these drugs improved the urine creatinine level by 47.83%, 76.40% and 65.20%, respectively. In case of creatinine clearance level, diabetic rats showed a significant decrease by 88.40% as compared with the control group. Diabetic rats prophylactically treated with rowatinex, melatonin or their combination showed significant increases by 490.00%, 495.00% and 706.80% as compared with the diabetic group with amelioration levels reached to 56.80%, 57.40% and 82.00%, respectively.

**Tab.1:** Protective effect of different drugs on kidney function parameters in rats with diabetic nephropathy

Groups	BUN (mg/dL)	Serum uric acid (mg/dL)	Serum creatinine (mg/dL)	Urine creatinine (mg/dL)	Creatinine clearance (mL/minute)
Control	26.48^f^±2.07	1.31^f^±0.07	0.42^g^±0.04	42.52^ab^±8.91	1.26^a^±0.23
Control Rowatinex	35.32^b^±2.02	1.68^e^±0.13	0.53^d^±0.05	42.94^ab^±7.76	0.992^c^±0.07
Control Melatonin	29.24^cde^±1.34	1.75 ^de^±0.109	0.44^fg^±0.06	41.28^ab^±11.43	0.89^c^±0.11
Control (Rowatinex+ Melatonin)	26.73^f^±1.48	1.64^e^±0.18	0.54^d^±0.06	40.84^b^±10.24	1.11^ab^±0.13
Diabetic	61.86^a^±2.02	3.89^a^±0.26	2.67 ^a^±0.18	13.86^d^±8.98	0.14^d^±0.04
Prophylactic (Rowatinex)	31.10^c^±2.22	2.04 ^c^±0.104	0.60^c^±0.06	34.20^c^±5.49	0.86^c^±0.09
Prophylactic (Melatonin)	26.68^f^±0.81	2.21^b^±0.09	0.48^ef^±0.05	46.38 ^a^±10.63	0.87^c^±0.10
Prophylactic (Rowatinex+ Melatonin)	36.66^b^±2.48	2.32 ^b^±0.14	0.53^d^±0.05	41.6^ab^±7.84	1.17^a^±0.82

• Data are mean±SD of ten rats in each group. • Statistical analysis are done using one way analysis of variance (ANOVA) using Co Stat Computer program accompanied with least significance level (LSD) between groups at *P *<0.05. • Unshared superscript letters are significant values between groups at *P*<0.0001.

Regarding on oxidative stress markers (***Table 2***), NO levels in diabetic rats showed a significant increase by 127.00% as compared with the diabetic group. Prophylactic rowatinex, melatonin or its combination significantly decreased NO level by 34.80%, 37.00% and 35.00%, respectively, as compared with the diabetic group. Therefore, protection of diabetic rats with these drugs improved tNO levels by 79.00%, 84.00% and 79.27%, respectively. In addition, malondialdehyde was significantly increased in diabetic rats by 55.20% as compared with the control group. Prophylactic rowatinex, melatonin or their combination on diabetic rats significantly decreased malondialdehyde contents by 28.00%, 23.00% and 22.00% with43.40%, 36.00% and 33.60% improvement, respectively. Contradictory, superoxide dismutase level was significantly decreased in diabetic rats by 35.00% vs. the control group. Diabetic rats prophylactically treated with rowatinex, melatonin and their combination showed significant increases in SOD level by 26.50%, 33.70% and 36.50% with 17.00%, 26.20% and 23.60% improvement, respectively. In addition, glutathione level significantly decreased by 31.00% in diabetic rats as compared with the control group. In diabetic rats prophylactically treated with rowatinex, melatonin and their combination, the glutathione levels were significantly increased by 26.30%, 20.50% and 31.70% with 18.00%, 14.60% and 22.20% improvement, respectively. Moreover, the total antioxidant capacity level showed a significant decrease by 46.40% in diabetic rats vs. the normal rats. Prophylactic diabetic rats showed significant increases of total antioxidant capacity level by 60.00, 68.50 and 48.00% upon prophylactically treated with rowatinex, melatonin or their combination, respectively, with 32.30, 37.00 and 26.00% improvement, respectively.

**Tab.2:** Protective effect of different drugs on oxidative stress markers in rats with diabetic nephropathy

Groups	NO (µmol/g tissue)	MDA (µg/mg protein)	SOD (µg/mg protein)	GSH (µg/mg protein)	TAC
Control	39.18^f^±3.95	70.68^g^±2.89	0.82^a^±0.07	2.21^a^±0.12	2.26^a^±0.176
Control Rowatinex	41.46^ef^±4.61	70.20^g^±5.04	0.77^b^±0.08	2.10^b^±0.22	2.05^b^±0.058
Control Melatonin	43.30^e^±6.8	73.20^g^±5.22	0.68^c^±0.06	1.77^f^±0.06	2.02^bc^±0.09
Control (Rowatinex+ Melatonin)	51.27^d^±13.09	74.54^g^±6.38	0.696^c^±0.06	2.01^a^±0.13	2.052^b^±0.13
Diabetic	88.92^a^±6.59	109.65^a^±7.50	0.53^d^±0.06	1.52^h^±0.057	1.21^g^±0.06
Prophylactic (Rowatinex)	57.94^c^±5.94	78.98^f^±6.76	0.67^c^±0.07	1.92^de^±0.09	1.94^de^±0.06
Prophylactic (Melatonin)	56.06^c^±4.98	84.48^e^±9.41	0.71^c^±0.08	1.84^ef^±0.11	2.046^b^±0.13
Prophylactic (Rowatinex+ Melatonin)	57.86^c^±4.12	85.90^de^±6.69	0.73^bc^±0.12	2.02^bcd^±0.22	1.79^f^±0.08

• Data are mean±SD of ten rats in each group. • Statistical analysis are done using one way analysis of variance (ANOVA) using Co Stat Computer program accompanied with least significance level (LSD) between groups at *P*<0.05. • Unshared superscript letters are significant values between groups at *P*<0.0001.

With respect to kidney injury molecule-1 (KIM-1) (***Table 3***), the diabetic rats showed a significant increase by 1,156.00% vs. the control group. This is in agreement with Chaudhary *et al. *[^23^] who reported that the early renal tubular damage biomarker including urinary KIM-1 were elevated in diabetes, even in those with normoalbuminuria. Prophylactic rowatinex, melatonin or their combination showed significant decreases in KIM-1 by 33.00%, 25.70% and 32.80% with 412.70%, 322.60% and 393.00% improvement, respectively. Treatment of diabetic rats with rowatinex, melatonin, or their combination, drug, and drug+ melatonin showed significant decreases in KIM-1 levels by 41.40%, 20.30%, 42.00%, 58.50% and 64.20% with 520.00%, 256.00%, 529.00%, 734.70% and 807.00% improvement, respectively.

**Tab.3:** Protective effect of different drugs on kidney injury indices in rats with diabetic nephropathy

Groups	KIM-1 (pg/mL)	HSP-70 (pg/100 mL)	Caspase-3 (pg/mL)	**TDF-**β (pg/mL)
Control	83.39^h^±22.5	1.80^i^±0.35	47.31^g^±11.60	115.24^h^±23.58
Control Rowatinex	97.36^h^±19.69	2.01^h^±0.23	38.60^gh^±3.99	125.16^h^±23.06
Control Melatonin	97.22^h^±21.03	1.68^i^±0.21	33.32^h^±3.45	88.91^I^±5.26
Control (Rowatinex+ Melatonin)	101.9^h^±26.84	2.29^g^±0.24	48.84^g^±3.31	114.24^h^±26.26
Diabetic	1047.5^a^±96.72	6.89^a^±0.46	362.76^a^±30.48	579.82^a^±5.09
Prophylactic (Rowatinex)	703.39^d^±26.08	4.84^d^±0.25	292.55^b^±4.41	471.00^c^±12.37
Prophylactic (Melatonin)	778.52^c^±17.54	4.76^d^±0.31	239.79^e^±17.46	426.38^e^±32.86
Prophylactic (Rowatinex+ Melatonin)	719.45^d^±10.39	4.844^d^±0.12	259.04^d^±5.05	438.72^e^±14.67

• Data are mean±SD of ten rats in each group. • Statistical analysis are done using one way analysis of variance (ANOVA) using Co Stat Computer program accompanied with least significance level (LSD) between groups at *P*<0.05. • Unshared superscript letters are significant values between groups at *P*<0.0001.

Regarding to HSP70, the diabetic rats' kidney showed a significant increase in its level by 282.70% as compared with the control group (***Table 3***). Prophylactic h rowatinex, melatonin or their combinationcaused significant decreases in HSP70 by 29.70%, 31.00%, and 30.30% with improvement levels amounting 113.80%, 118.00% and 114.00%, respectively. Treatment of diabetic rats with rowatinex, melatonin, their combination, drug, and drug+ melatonin showed significant decreases in HSP70 level by 24.50%, 16.50%, 29.50%, 53.40% and 60.00% with 93.30%, 63.30%, 112.20%, 205.00% and 228.30% improvement, respectively.

The protein contents in the diabetic rats' kidney showed a significant decrease by 37.30% as compared with the control group (***Table 4***). Conversely, the total serum protein contents in diabetic rats showed a significant increase by 71.40% as compared with the normal control group. Prophylactic rowatinex, melatonin or their combination showed significant decreases by 60.50%, 40.00% and 46.28% as compared with the diabetic group, with 104.00%, 68.50% and 79.30% improvement, respectively.

Serum caspase-3 level in diabetic rats was significantly increased by 667.00% as compared with the control group (***Table 3***). Prophylactic rats with rowatinex, melatonin or their combination significantly decreased serum capase-3 by 19.30%, 33.90% and 28.60% comparing with the diabetic groups, with 148.00%, 260.00% and 219.00% improvement, respectively.

Serum TGFβ1 was significantly increased by 403.00% as compared with the control group (***Table 3***). Prophylaxis with rowatinex, melatonin or their combination caused significant decreases by 18.76%, 26.50% and 24.30% as compared with the diabetic groups, with 94.40%, 133.00% and 122.00% improvement, respectively.

**DNA fragmentation and histopathologic features **

**Fig.1:**
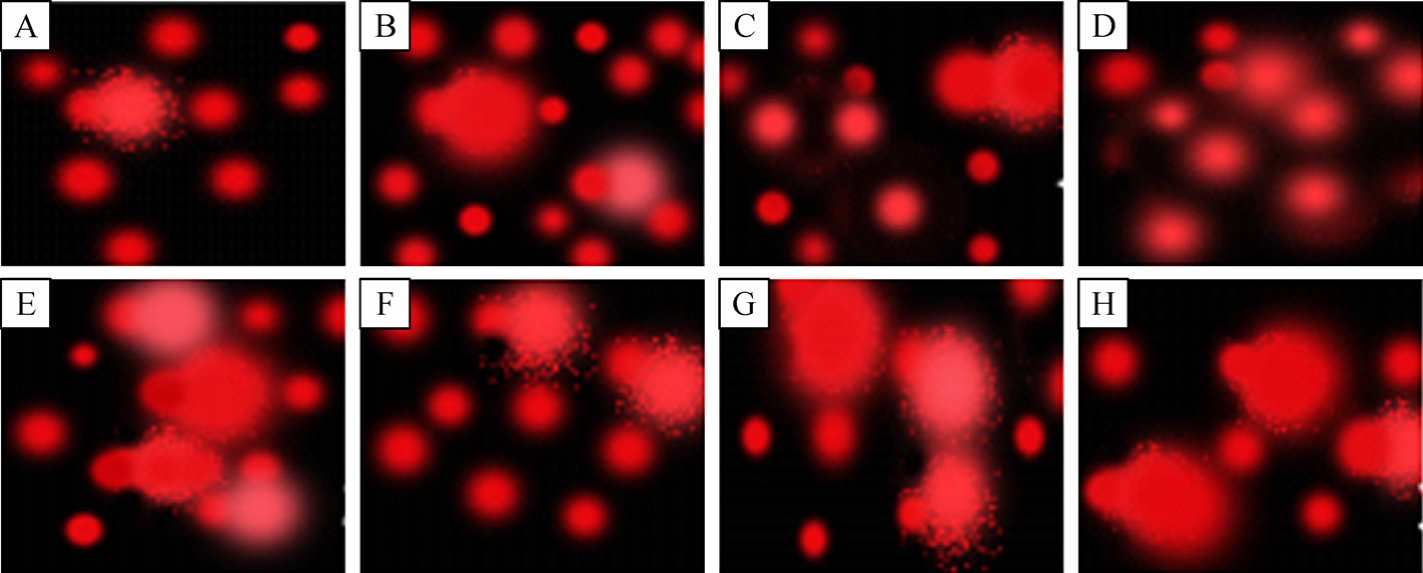
Electrophoretic pattern of DNA fragmentation in the renal tissues of diabetic rats. Control rats received normal saline (A), melatonin (B), rowatinex (C), or melatonin plus rowatinex (D), and diabetic rats received normal saline (E), prophylactic melatonin (F), rowatinex (G), or melatonin plus rowatinex (H). Minimal breakage and movement of DNA are seen in diabetic rats prophylactically treated with melatonin.

Regarding to the DNA fragmentation, DNA tail length in rats diabetic kidney was significantly increased by 25.30% (***Table 4*** and ***Fig. 1***). Prophylaxis with rowatinex, melatonin or their combination showed significant decreases by 28.30%, 25.00% and 32.20% as compared with the diabetic groups, with 100.00%, 88.50% and 114.00% improvement, respectively. The percentage of tailed DNA numbers in diabetic rats reached to 245.00% as compared with the control group. Prophylactic rowatinex, melatonin or their combination showed significant decreases in tailed DNA by 25.00%, 19.40% and 27.40%, with 86.00%, 67.00% and 94.60% improvement, respectively. Significant increase in the tail-moment (1,069.00%) in diabetic rats was recorded as compared with the control group. Prophylactic diabetic rats with rowatinex, melatonin or their combination showed significant decreases in the unit-tail (moment) by 46.00%, 40.50% and 53.00% with 540.00%, 474.00% and 619.00% improvement, respectively.

**Tab.4:** Protective effect of different drugs on DNA degradation and protein contents in rats with diabetic nephropathy

Groups	DNA tail length (µm)	% of tailed DNA	Tail moment (Unit)	Tissue total protein (mg/g)	Serum total protein (g/dL)
Control	1.502^i^±0.49	1.492^j^±0.33	2.35^I^±1.22	8.14^abc^±0.81	6.05^cd^±0.44
Control Rowatinex	1.89^g^±0.08	2.14^h^±0.022	4.014^h^±0.18	6.76^d^±0.84	6.18^cd^±1.052
Control Melatonin	1.682^h^±0.25	1.73^i^±0.22	3.016^I^±0.84	7.81^bc^±0.58	5.46^d^±0.38
Control (Rowatinex+ Melatonin)	2.23^f^±0.36	2.45^g^±0.30	5.31^g^±1.51	8^abc^±0.58	6.83^bc^±0.28
Diabetic	5.30^a^±0.21	5.14^a^±0.31	27.47^a^±2.74	5.10^e^±0.48	10.37^a^±3.20
Prophylactic (Rowatinex)	3.816^b^±0.17	3.86^cd^±0.067	14.80^c^±0.59	8.52^a^±0.64	4.09^e^±1.23
Prophylactic (Melatonin)	3.97^b^±0.18	4.14^b^±0.036	16.33^b^±0.86	8.40^ab^±0.43	6.22^cd^±0.96
Prophylactic (Rowatinex+ Melatonin)	3.59^c^±0.13	3.73^d^±0.48	12.92^d^±0.577	7.74^c^±0.70	6.05^cd^±0.44

• Data are mean±SD of ten rats in each group. • Statistical analysis are done using one way analysis of variance (ANOVA) using Co Stat Computer program accompanied with least significance level (LSD) between groups at *P*<0.05. • Unshared superscript letters are significant values between groups at *P*<0.0001.

**Fig.2:**
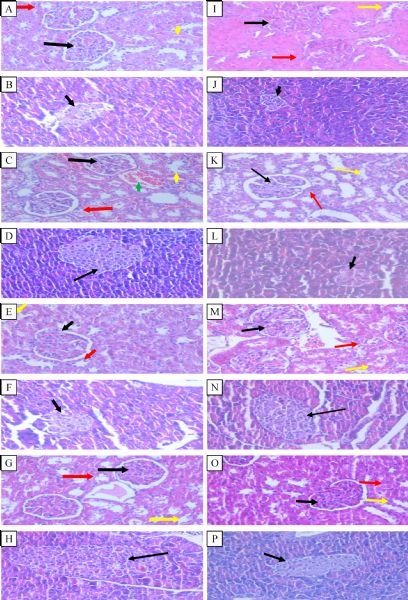
**Histopathological sections of rats kidney and pancreas **A: Kidney section of control rats shows the renal cortex of the renal corpuscle with normal glomeruli (black arrow), normal pattern of proximal (red arrow) and distal convoluted (yellow arrow) tubules. B: Pancreatic section of control rats shows atrophic pancreatic islets of Langerhan's as shown by arrow. C: Kidney section of control rats treated with rowatinex shows the renal cortex of the renal corpuscle with normal glomeruli (black arrow), normal pattern of proximal (red arrow) and distal convoluted tubules (blue arrow) with congested blood vessels (green arrow). D: Pancreatic section of control rats treated with rowatinex drug shows regular pancreatic islets of Langerhans as shown by arrow. E: Kidney section of control rats treated with melatonin shows the renal cortex of the renal corpuscle with mild cellularity, mild obliterated capsular space (black arrow), mild thick wall of the proximal tubules (red arrow) and distal convoluted tubules (yellow arrow). F: Pancreatic section of control rats treated with melatonin shows normal size pancreatic islets of Langerhan's with regular shape as shown by arrow. G: Kidney section of control rats treated with rowatinex and melatonin shows the renal cortex of the renal corpuscle with normal glomeruli (black arrow), normal pattern of the proximal (red arrow) and distal convoluted tubules (yellow arrow). H: Pancreatic section of control rats treated with rowatinex and melatonin shows hypertrophy of β-cells of islets of Langerhan's as shown by arrow. I: Kidney section of diabetic rats shows the renal cortex with corpuscles of high cellularity and obliterated capsular space (black arrow). The proximal and distal convoluted tubules show disrupted epithelia (red and yellow arrow). J: Pancreatic section of diabetic rats shows atrophic pancreatic islets of Langerhan's as shown by arrow. K: Kidney section of diabetic rats prophylactically treated with rowatinex shows the renal cortex of the renal corpuscle with normal glomeruli (black arrow), normal pattern of the proximal (red arrow) and distal convoluted (yellow arrow) tubules. L: Pancreatic section of diabetic rats prophylactically treated with rowatinex shows atrophic pancreatic islets of Langerhan's, as shown by arrow. M: Kidney section of diabetic rats prophylactically treated with melatonin shows the renal cortex of renal corpuscle with moderate cellularity and mild obliterated capsular space (black arrow), thick wall of the proximal (red arrow) and convoluted (yellow arrow) tubules. N: Pancreatic section of diabetic rats prophylactically treated with melatonin shows almost normal size pancreatic islets of Langerhan's with regular shape as shown by arrow. O: Kidney section of diabetic rats prophylactically treated with rowatinex and melatonin shows the renal cortex of the renal corpuscle with moderate cellularity, mild obliterated capsular space (black arrow) and normal pattern of the proximal (red arrow) and distal convoluted (yellow arrow) tubules. P: Pancreatic section of diabetic rats prophylactically treated with rowatinex and melatonin showed regular pancreatic islets of Langerhan's as shown by arrow (H&E, ×400). A representative photograph is shown from each group (*n* = 5).

DNA fragmentation in diabetic rats using comet assay technique is illustrated in ***Fig. 1***. Prophylactic rowatinex, melatonin or their combination decreased DNA fragmentation by 4.20%, 40.50%, and 51.80% as compared with the diabetic groups, with 42.00%, 40.50% and 32.40% improvement, respectively. Regarding to the histopathologic analysis of the kidneys and pancreases, control rats and control treated with rowatinex, melatonin and their combinations showed more or less normal kidney and pancreatic architectures (***Fig. 2***). The renal cortex of diabetic rats showed high cellularity and obliterated capsular space of corpuscles. The epithelia were disrupted in the proximal convoluted tubules. Pancreatic section from diabetic rats showed atrophic pancreatic islets of Langerhan's (***Fig. 2***).The kidney of diabetic rats prophylactically treated with rowatinex showed enhancement to the normal state, while the pancreas showed moderate enhancement. Melatonin and the combination of melatonin and rowatinex showed moderate improvement in kidney and normal appearance of pancreas architecture (***Fig. 2***).

## LangerhanDiscussion

Diabetic nephropathy is a progressive kidney disease caused by angiopathy of capillaries in the renal glomeruli. It is characterized by nephrotic syndrome and diffuse glomerulosclerosis due to long standing diabetes mellitus. Loss of body weight and high blood glucose level were observed in our study. These observations are in accordance with the reports of Zafar and Naqvi [^5^] and Pi* et al.* [^24^]. Zafar and Naqvi [^5^] attributed the decrease in body weight to the alkylation of DNA by streptozotocin that produced hyperglycaemia and necrotic lesions, while Pi *et al. *[^24^] attributed the increase in glucose level to the deficient antioxidative capacity of the pancreatic β-cells that makes them highly susceptible to oxidative changes. The cell damage by free radicals leads to β-cell degranulation or necrosis, resulting in a decrease in insulin secretion and an increase in blood glucose level. Kanter *et al. *[^25^] showed that melatonin (50 mg/kg) treatment caused a sharp decline in serum glucose, a slight increase in serum insulin concentrations and partial regeneration/proliferation of islet β-cells. This was attributed to the immunostimulatory effect of melatonin, which stimulated the secretion of opioid peptides and cytokines from lymphocytes and prevented the translocation of nuclear factor-kB to the nucleus and its binding with DNA [^26^]. The reduction of hyperglycemic state with melatonin treatment helps to reduce the severity of inflammation in diabetes.

In parallel with our results, Parving *et al.* [^27^] recorded disturbance of kidney function indices in streptozotocin-induced type 2 diabetic rats with severe microalbuminuria. Thorsten [^12^] attributed the improvement of such parameters after rowatinex treatment to the presence of terpenes. Anwer [^28^] postulated the role of melatonin as antioxidant agent that attenuated the severe effects of type 2 diabetes by attenuating hyperinsulinemia, glucose intolerance and insulin resistance.

It is well known that inflammation and oxidative stress play important roles in podocyte injury and the downregulation of nephrin and podocin [^29^] indicating severe nephritis. The observed disturbance in the antioxidant parameters indicated increased oxidative stress, suggesting their role in the kidney response to inflammatory stimuli [^30^]. Treatment with melatonin and rowatinex as antioxidant agents reversed the changes of the oxidative stress markers.

Vaidya *et al.* [^31^] mentioned that the regression of diabetic nephropathy severity after treatment was associated with reduction of urinary KIM-1, which confirmed our results. Barutta *et al. *[^32^] found that diabetes expressed HSP70 in the glomeruli and in the medulla, which may explain the ability of renal cells to increase the effectiveness of cytoprotective response. As diabetes has oxidative properties and HSP70 is well expressed, treatments with the selected drugs improve the level of HSP70 through their antioxidative effects and the resulted protein depression.

Caspase-3 is one of the effector caspases of the apoptotic pathways. Apoptosis has been demonstrated to mediate cell death in a variety of renal diseases, including diabetic nephropathy. Frances* et al.* [^33^] demonstrated that apoptosis in streptozotocin-induced diabetic rats was associated with elevation of OH that contributed partially to mitochondrial cytochrome c release and elevation of caspase-3 activation. Recently, several studies have described the involvement of macrophages in the pathogenesis of diabetic nephropathy. In line with this observation, we speculate that infiltrating macrophages stimulate mesangial cells to secrete TGFβ1 [^34^]. Frances* et al. *[^33^] reported that treatment of diabetes attenuated free radical elevation which improved the apoptotic condition.

The comet assay technique in the present study is a commonly used method to detect oxidative damage in lymphocyte DNA and the degree of oxidative stress is related to the size of comet tail [^35^]. The decrease in protein content may be due to a decrease in ribosomal granules of rough endoplasmic reticulum or due to a decrease in DNA content. The decrease of DNA content was associated with a decrease in protein content in kidney cells and pancreas of diabetic rats [^36^].

We observed that melatonin showed the most potent effect in most of the biochemical selected parameters followed by the combination of rowatinex and melatonin, revealing a synergic effect through the observed highly improved level in each parameter. The histopathologic observations are in parallel with the finding of Eliakim-Ikechukwu and Obri [^37^] and confirmed our biochemical results.

In conclusion, melatonin protects against kidney injury associated with type 2 diabetes mellitus in rats followed by its combination with the naturally occurring drug rowatinex. Rowatinex alone improves the kidney histopathologic features, while melatonin improves the pancreas architecture.
